# Challenges faced by women with disabilities in accessing sexual and reproductive health in Zimbabwe: The case of Chitungwiza town

**DOI:** 10.4102/ajod.v6i0.252

**Published:** 2017-05-26

**Authors:** Tafadzwa Rugoho, France Maphosa

**Affiliations:** 1 Livelihoods Programme, Leonard Cheshire Disability Zimbabwe, Zimbabwe; 2 Department of Sociology, University of Botswana, Botswana

## Abstract

**Background:**

Women with disabilities in Zimbabwe face numerous challenges in accessing sexual and reproductive health. Cultural belief still regards them as not sexually active. The government has also failed to promote policies that facilitate access to sexual and reproductive services by women with disabilities.

**Objectives:**

The reseach objectives were to explore the challenges faced by women with disabilities in accessing sexual and reproductive health in Zimbabwe.

**Method:**

The data were gathered using in-depth interviews with 23 purposively selected respondents. Thirteen women had physical disabilities, five were visually impaired, three were deaf and two were stammering. The respondents with physical disabilities were using wheelchairs, walking frames, prosthesis, crutches and caliper shoes. The participants’ ages ranged from 18 to 45 years. All interviews were transcribed and translated verbatim into English, and passages were extracted from the transcripts. Key themes and concepts were identified and coded to offer a rich framework for analysis, comparisons and presentation of the data.

**Results:**

Negative perceptions of health personnel towards people with disabilities, disability-unfriendly infrastructure at health facilities and absence of trained personnel for people with disabilities (sign language) are some of the challenges involved.

**Conclusion:**

The government, in partnership with other stakeholders, should address challenges faced by women with disabilities when accessing sexual and reproductive health services. Non-government, private hospitals and profit-making organisations should join hands with government in funding health requirements for women with disabilities.

## Introduction

There has been global attention on sexual and reproductive health recently. The United Nations Convention on the Rights of Persons with Disabilities (UNCRPD) which became part of international law stipulates that governments should guarantee access to sexual reproductive health to people with disabilities (United Nations 2007). Global studies prove that women with disabilities still face a plethora of challenges in accessing sexual and reproductive health services (Boezaart 2012). Institutionalised discrimination, isolation and stereotyping of women with disabilities continue unabated (Rugoho & Siziba 2014). Violations of the sexual and reproductive rights of the women with disabilities have been condoned in developed and developing nations. Governments and development partners, especially in developing countries, have failed to offer affordable and accessible sexual and reproductive health facilities (Groce et al. 2009; Swartz et al. 2009). Women with disabilities are still viewed as people who cannot take part in sexual and reproductive activities as observed by Swartz et al. (2009) and Groce et al. (2009). Negative attitudes towards sexual and reproductive rights of women with disabilities still exist. Women with disabilities are still perceived as non-sexual or as not having the capacity to engage in sexual activities (Chikumbu 2014). That they are viewed as broken objects has made their plight remain on the periphery of policymakers (Choruma 2007).

The population of persons with disabilities is estimated to constitute 15% of the world population (WHO 2011). It is further estimated that 19% of women with disabilities are domiciled in Third World countries. These women constitute three-quarters of the women living in absolute poverty globally. These women are excluded from economic empowerment initiatives on account of their gender and their disability (Rugoho & Siziba 2014). Women with disabilities are more prone to sexual abuse and victimisation because they are considered to be weak and hence easy targets (Rugoho & Maphosa 2015; Shuttleworth 2007). The sexual rights of women with disabilities are further compromised by factors such as negative attitudes of family and society, and cruel religious and cultural practices (Rugoho & Maphosa 2015). The negative attitudes also cascades to health providers and medical staff (Bath 2008; Burgen 2010).

The UNCRPD sexual and reproductive rights have become part of the fundamental human rights (UN 2007). These rights are also recognised under the Constitution of Zimbabwe, which guarantees everyone his or her sexual and reproductive rights (Government of Zimbabwe [GoZ] 2013). Under the UNCRPD, women with disabilities are also provided the opportunity to start their own families without interference from family or the state.

## Problem statement

Zimbabwe still faces challenges in the provision of sexual and reproductive health services. In an effort to address challenges in sexual and reproductive health, the National Reproductive Health Policy of Zimbabwe (2006) was developed by the GoZ. However, the sexual and reproductive needs of women with disabilities were not captured in the policy. As noted by Choruma (2007), the needs of persons with disabilities, including women with disabilities, still remain a peripheral issue. People with disabilities continue to be treated as second-class citizens (Rugoho & Siziba 2014). Women with disabilities face barriers in accessing sexual and reproductive health services. It is against this background that the study sought to understand the challenges faced by women with disabilities in accessing sexual and reproductive health services in Zimbabwe.

## Research objectives

The main aim of the study was to explore the challenges faced by women with disabilities in accessing sexual and reproductive health in Zimbabwe using Chitungwiza as a case study. The study was guided by the following specific objectives:

- To examine the challenges faced by women with disabilities in trying to access sexual and reproductive health.
- To find sources of information on sexual and reproductive health accessible to women with disabilities.
- To explore strategies that can be adopted to improve sexual and reproductive health for women with disabilities.

## Literature review

Accessing sexual and reproductive health has become a fundamental right in the 21st century, and this has also posed a global challenge (Mprah 2013). Africa and other parts of the Third World are battling to provide sexual and reproductive services due to other pressing issues (Glasier et al. 2006). Women with disabilities have been noted by the World Health Organization (WHO) to be the most disadvantaged and alienated group when it comes to accessing sexual and reproductive health services (WHO 2009). The WHO noted that the chief challenge is the community’s negative attitudes towards people with disabilities, which have been institutionalised and have caused untold pain to women with disabilities. Prejudice, stereotyping and discrimination against people with disabilities have resulted in serious violations of their sexuality and reproductive rights. Practices such as coerced sterilisation and forced administration of lifelong contraceptives are still performed on women with disabilities without their consent. Women with mental disabilities are more prone to forced abortions and sterilisation (Mykitiuk & Chadha 2011). In most documented cases, relatives decided on behalf of women with disabilities without their consent (Ouellette 2008).

In Africa, including Zimbabwe, people with disabilities still continue to be treated as second-class citizens (Rugoho & Siziba 2014). They are not expected to indulge in sexual activities (Chikumbu 2014). Rugoho and Maphosa (2015) found that African communities perceive people with disabilities as hypersexual. Discussing with them sexual and reproductive issues would trigger their sexual feelings and they would not be able to control their sexual desires. However, for Hunt and De Mesquit (2006), European societies feel pity and sorry for people with disabilities and often conclude that their physical appearance would not allow them to have sexual intercourse. They are viewed as sick people who need to heal first before they could indulge in sexual activities. Sexual activities would harm them and further disable them. Mgwili & Watermeyer (2006) further note that South African communities view people with disabilities as not having enough mental stamina to start or be involved in any meaningful sexual relationships. Women with disabilities are often thought of as not being strong enough to carry pregnancies as observed by Hunt and De Mesquit (2006). Blackburn (2002) argues that communities do not have enough knowledge and information about disability issues. The challenges faced by women with disabilities in accessing sexual and reproductive health services are multifaceted; they are caused by economic, political, cultural and educational factors.

WHO (2013) and Groce et al. (2009) conclude that women with disabilities need greater access to sexual and reproductive health services than their able-bodied counterparts. Their disability also increases their vulnerability to sexual abuse (Rugoho & Maphosa 2015). Groce et al. (2009) found enough evidence to conclude that women with disabilities are three times more likely to be victims of sexual, emotional and physical abuse. With all that compelling evidence, governments have not formulated policies to increase access to sexual and reproductive health for women with disabilities (Groce & Trasi 2004). Studies conducted by Job (2004) and Prilleltensky (2004) found out that adolescents with disabilities are not given the opportunity to learn about sexual and reproductive health as compared with their peers because teachers, parents and counsellors fear to discuss sexual and reproductive health with them because they are perceived to be non-sexual. As such, they miss out on basic vocabulary to describe changes in their bodies (Groce, Yousafzai & Maas 2007; WHO 2009). Deaf women and women with physical disabilities face similar challenges (WHO 2009). Development of literature in Braille and other formats is still a challenge in developing countries. Roberts (2006) notes that deaf women are usually not given proper information owing to challenges in conversing in sign language. Fedorowicz (2006), Heyederick (2006), Wilson & Monaghan (2006) and Groce et al. (2007) also observe that there is little literature available for deaf women in the area of sexual and reproductive health. Medical staff in developing countries are usually not trained in sign language and often find it difficult to communicate with deaf women when they visit health centres (Margellos-Anast, Estarziau & Kaufman 2006).

The marginalisation of women with disabilities in sexual and reproductive health services presents a challenge in the global fight against HIV and AIDS. Initiatives and policies that embrace the sexual and reproductive health of women with disabilities are essential in fighting the spread of HIV and AIDS (Bankole & Malarcher 2010). Sexual and reproductive health needs of women with disabilities need to be seriously taken on board by governments. WHO (2004) also notes the physical barriers that prevent women with disabilities from accessing sexual and reproductive health. Such barriers include lack of clear directions and services on offer, crowding and lack of privacy. Mulumba et al. (2014) also observed similar challenges among women with disabilities in Uganda. In Uganda, Ahumuza et al. (2014) found that the negative attitudes of health care providers made it difficult for women with disabilities to access sexual and reproductive health services. Health staff would use abusive and insulting language when dealing with women with disabilities who were pregnant. They often assume that women with disabilities are not sexually active. Lack of confidence, shyness, poor relations with health staff and low literacy levels are some of the challenges encountered by women with disabilities in Cambodia (Senderowitz, Hainsworth & Solter 2003). Holness (2013) also found that women with disabilities still continue to face prejudice and discrimination daily. This has negatively affected their access to sexual and reproductive health services. In South Africa, the practice of forced sterilisation is still rampant. Holness (2013) cites court cases that prove that women with disabilities have been forced to under go sterilisation. Human Rights Watch (2013) argues that involuntary sterilisation done on women with disabilities is an act of violence, and it is degrading to the human being. Canadian sexual and reproductive health policies discourage women with disabilities from participating in procreation activities (Gibson & Myklitiuk 2011). In Canada, it is largely assumed that women with disabilities will transfer their disabilities to the unborn child; hence, to break this circle of having children with disabilities women with disabilities are discouraged from giving birth. They are often assumed to have no capacity to take care of the children. Court cases in America and Britain also prove that forced sterilisation among women with disabilities exists in those countries (Rioux & Patton 2011). In Australia, research has demonstrated that forced sterilisation still exists, and in many such cases, consent is given by parents (Brady, Briton & Grover 2001).

In 2007, the world made a bold statement in trying to promote the rights of people with disabilities. The world saw the birth of UNCRPD, which sought to address all the injustices being faced by people with disabilities (UN 2007). The convention gave elaborate rights to people with disabilities, including accessing sexual and reproductive health. The right to sexual and reproductive health was clearly articulated in Article 23, which is well supported by Article 12 which established that people with disabilities have to be recognised everywhere as persons with standing before the law. They should also enjoy equal and just treatment. The Convention on the Rights of Persons with Disabilities (CRPD) further guarantees that women with disabilities should be given a voice in determining their own medical choices. It further protected them from forced sterilisation. The CRPD recognised that forced sterilisation and forced abortion for women with disabilities without their consent amounts to violation of their human rights. The Committee on the Elimination of Discrimination against Women (CEDAW) in 1999 also outlawed the practice of forced sterilisation of women with disabilities. This was further clarified by the same committee in 2010 when it declared that, regardless of whether women have disabilities or not, sterilisation should not be done without informed consent (CEDAW Committee 2010)

In Zimbabwe, sexual and reproductive health issues came to the limelight in 2006 when the government formulated the National Reproductive Health Policy. The policy offers services such as maternal health, family planning, treatment for sexually transmitted diseases including HIV and AIDS and adolescent reproductive health. Surprisingly the policy proffered few interventions towards women with disabilities. One of the main reasons for this omission is that disability issues are still regarded as charity issues; hence, funding for sexual reproductive health of women with disabilities is still a challenge (Kabzems & Chimedza 2002). Very few studies have been done in Zimbabwe on sexual and reproductive health among women with disabilities; hence, very little is known about the challenges they face in accessing sexual and reproductive health. Much research on sexual and reproductive health has focused on youth and people with HIV and AIDS (Wilcher & Cates 2009). In Uganda, also a developing country, some policy frameworks have been in place to include and mainstream disability issues across sectors (Khumalo 2008; Lang 2009). In Zimbabwe, a number of non-government organisations such as Leonard Cheshire Disability Zimbabwe, Disability, HIV and AIDS Trust and Deaf Zimbabwe Trust are working with women with disabilities to help them access sexual and reproductive health. However, they are limited to advocacy and awareness-raising issues.

## Methodology

### Study site

The study was conducted in Chitungwiza town which is about 25 kilometres from Harare, the capital city of Zimbabwe. Chitungwiza was chosen because it is a densely populated town which also houses a number of organisations for people with disabilities. In selecting respondents, a woman with disabilities was considered the focal person as she had already established a rapport working with disabled women in Chitungwiza. She was also an expert in sign language. She was also knowledgeable about the area.

### Study design and sample

In gathering data, the researchers adopted a qualitative research design. The data were gathered using in-depth interviews with 23 purposively selected respondents. Thirteen women had physical disabilities, five were visually impaired, three were deaf and two were stammering. The respondents with physical disabilities were using wheelchairs, walking frames, prosthesis, crutches and caliper shoes. The participants’ ages ranged from 18 to 45 years. The study specifically targeted women who were older than 18 years of age because at age 18 they reached the age of consent, which gave them the right to consent to sexual activities and to make their own choices about sexual and reproductive health issues. At this age, they would have the right to consent to such a study. Seven participants were married while 13 were single mothers and 4 were not yet married but were in sexual relationships. Out of the 17 respondents who were not married, 8 of them reported that they were staying on their own and 9 reported to be staying with relatives. The languages used in the research were Shona, a local language which was understood by many participants, and sign language for the three deaf participants. All participants were able to read and write. They had all completed primary education.

### Data collection and processing

In-depth interviews were deemed to be the most suitable data collection method as this study required detailed accounts of the subjective experiences of women with disabilities in accessing sexual and reproductive health services.

Data collection lasted three weeks. A dicta-phone was used to record all the interviews. The researchers also took handwritten notes. The interviews were digitally recorded and field notes were taken during the interviews with the consent of the respondents. Each interview session lasted between 45 minutes and 1 hour. All interviews were transcribed and translated verbatim into English, and passages were extracted from the transcripts. Key themes and concepts were identified and coded to offer a rich framework for analysis, comparisons and presentation of the data. The respondents’ individual experiences, comments and opinions were then categorised according to recurring selected themes from all the interview transcripts.

### Ethical considerations

The researchers were aware that sexual and reproductive health and disability are sensitive subjects and therefore observed and adhered to strict ethical conduct throughout the study. Before carrying out the interviews, preliminary meetings and telephonic discussions were held with the prospective participants where the researchers explained the nature and purpose of the study and informed respondents that their participation was entirely voluntary and it was within their rights to withdraw from the study at any time without having to give any explanation. The prospective respondents were also assured that the information they would give would be treated with strict confidentiality and that they would remain anonymous. They were informed that information would be used only for academic purposes and not for any other purposes. The use of Shona, which is a local language, ensured that all respondents were very conversant and that there were no ambiguities in communication. It made it easy for the respondents to share their experiences.

## Results

The study found that women with disabilities experience a number of challenges in accessing sexual and reproductive health services in Chitungwiza. The following sections discuss the barriers identified.

### Attitudinal barriers

Negative attitudes towards women with disabilities made it difficult for them to visit health centres to seek information and services on their sexual and reproductive health. Female nurses were cited as major culprits in insulting women with disabilities when they visit hospitals when they are pregnant or present for treatment. This was explained by a 33-year-old mother with physical disabilities of four children and working as a vendor who said:

‘I visited the clinic when I was pregnant with my fourth child. The nurses said very hurtful things to me. They said I was giving birth like a dog. They said they pitied the men who introduced me to sex because I was no longer able to control my sexual feeling. I will never go back to that clinic again.’

Similar sentiments were also expressed by another 29-year-old pre-school teacher, visually impaired, who said:

‘I went to look for family planning methods at the clinic and the nurses told me that sex was not meant for the disabled, hence there was no need for me to get contraceptive methods.’

### Physical barriers

Many clinics and hospitals are located far away from the residences of many of the respondents. Some claimed that they had to walk long distances to get to the nearest clinic. For those who rely on personal aid for mobility, the process of accessing the nearest health facility was expensive because on public transport they had to pay for two people – themselves and their assistants. In some cases, they pay for their wheelchairs as well. Inaccessible buildings and facilities were also cited as impediments to access sexual and reproductive services. Many health centres in Zimbabwe, including those in Chitungwiza, were not constructed with people with disabilities in mind. A 39-year-old woman who injured her spine in a car accident and is now using a wheelchair for mobility said:

‘The clinics do not have ramps to help those on wheelchairs like me. One day I decided to go to the clinic to ask for information on sexual and reproductive health. I had problems negotiating my way around the buildings. When I asked for help the nurses told me that they could not help and that I should have come with my relatives to aid me. I was so humiliated and frustrated that I developed a headache for which I ended up getting treatment and forgot about the information on sexual and reproductive health.’

The respondents also cited toilets which are always dirty and not user-friendly for people with disabilities as another challenge they face when they visit health centres to seek sexual and reproductive health services. With Zimbabwe facing a water crisis, hygiene is often compromised. Many people have died in the country from cholera and related diseases. Dirty toilets at public health institutions present a special challenge to women with disabilities. This point was aired by a 42-year-old female participant who uses a wheelchair for mobility.

‘The toilets are a health hazard … you can die from using those toilets. They are always dirty and that makes life very difficult for people with disabilities. Can you believe that I had to step on human faeces and urine with my wheelchair for me to reach the toilet seat? I got diarrhoea a few days after that and I suspect I got it from contaminated hands.’

A 20-year-old visually impaired female participant also stated:

‘How does one expect a blind person like me to use a dirty toilet with human faeces and urine flowing everywhere? I once stepped on human faeces and was unaware of it, only to be told by other people. It is so embarrassing.’

A secondary schoolgoing 19-year-old participant who was paraplegic and used crutches for mobility also had this to say:

‘Sometimes patients are sometimes asked to bring their own water if they want to use the toilets. No water, no toilet! This presents a big challenge for women with disabilities who are unable to carry water containers because of the nature of their disabilities.’

### The cost of services

Respondents stated that health centres, including those that are state owned, and local authorities charge consultation fees of amounts ranging from US$5–20. Besides the consultation fee, patients also have to pay for the services and supplies they receive. Before the economic meltdown that began in 2000, women with disabilities used to receive social grants from the disability fund administered by the Ministry of Social Welfare. The disbursement of the grant has become increasingly erratic with some beneficiaries not having received any payment for the past 10 years. Participants highlighted that their inability to pay user fees had limited and in some cases entirely curtailed their visits to medical facilities.

Another 24-year-old female participant with physical disabilities who stayed with relatives said:

‘They want us to pay ten dollars consultation fees at clinics and hospitals. Where do they think we will get such money from? I am not employed. I stay with relatives and it is difficult for me to ask them for money to go and seek reproductive services because they are already taking care of a lot of my needs.’

She was supported by another 20-year-old single female visually impaired participant:

‘Many disabled women are not employed. Employers in government and the private sector are reluctant to give jobs to disabled women, yet hospitals are not ready to treat us without paying. It’s not that I am against the idea of paying for sexual and reproductive health services. Some of us simply cannot afford it. We have other needs such as accommodation and food and all the little money that we get is even barely enough for those basic needs. When one is pre-occupied with meeting basic daily needs, going to a hospital to pay for sexual and reproductive services receives very low priority.’

### Lack of privacy

Privacy for women with disabilities was also mentioned as a deterrent among these women while visiting health centres. The participants felt that their privacy was often violated by health staff. This was pointed out by a 21-year-old visually impaired participant working as as a beggar in town who stated:

‘I developed a boil close to private genitals and I went to the hospital. During the examination with the nurse at the hospital other people had access inside the examination room. They think just because I am blind I did not need privacy. I could hear their footsteps as they were getting in and out of the room. It made me uncomfortable. Sometimes other nurses would come and they would start discussing about me.’

This was also corroborated by another 29-year-old visually impaired woman who is employed as a teacher by a private school:

‘When you are blind, they don’t care about privacy.… When I was pregnant, I would be examined by dozens of people, including non-medical personnel in the hospital because the nurses would invite their friends in the consultation room.’

Respondents pointed out that health workers did not know how to relate to people with disabilities, especially in the presence of their helpers. Most often they communicate to the person with a disability through the third person instead of communicating directly with the person concerned. This limits the extent to which women with disabilities could freely share confidential sexual and reproductive health information with health workers. This sentiment was expressed by a 27-year-old woman amputee who uses a wheelchair:

‘The last time I visited the clinic the nurses were asking my friend about what I wanted instead of directing the questions to me. I felt they treated as a child because I am disabled. I felt humiliated because my friend ended up knowing about the condition that I was seeking treatment for.’

Another 19-year-old female, who uses a wheelchair and participates in wheelchair baske ball, had this to say:

‘They ended up discussing with my mother the most suitable family planning method without me. I was left seated as a zombie while they discussed issues that concerned my health.’

### Lack of sign language

Women with deafness highlighted that their biggest challenge was nurses who understood sign language. In Zimbabwe, most of the professionals do not have sign language training. This prevents deaf people from getting enough and relevant information on sexual and reproductive health. A 26-year-old deaf female participant who had five children said:

‘For me, I think the doctors and nurses always guess what we are saying. They can’t talk to us and how do they expect to help us. It’s a waste of time because of the language barrier.’

This was supported by another 35-year-old deaf person staying with relatives and working as a voluntary sign language teacher who asserted that:

‘If you are deaf, they communicate with you through your interpreter. The problem with this is when one wants to discuss issues that are so sensitive and confidential with the nurse that one does not want the interpreter to know.’

Another 28-year-old deaf participant with a university degree said:

‘I was made to write what I wanted. Then they complained that they could not understand what I had written. The whole thing ended up being a drama because more than five nurses were called to help. I felt very embarrassed and I do not wish to visit that hospital again.’

Women with disabilities also felt that they were not being given enough attention by doctors and nurses when they visited the hospital and clinics. A woman with a stammering problem stated that owing to the large numbers of people they have to deal with, doctors and nurses get impatient with people who have speech problems. As a result, people with speech disabilities do not get the opportunity to fully explain their problems.

### Restricted access to information

The participants indicated that women with disabilities obtained information on sexual and reproductive issues from various sources, including health institutions, schools, parents, social media and peers. However, most of these sources of information have aspects that make them inaccessible to women with disabilities. People with disabilities in Zimbabwe do not have equal chances of going to school as their able-bodied counterparts; hence, they do not get a chance to be taught about sexual and reproductive health in schools. Parents often do not educate their children with disabilities on sexual and reproductive issues.

Social media, which has become a popular and cheap way of getting information about various issues, has offered women with disabilities a platform to discuss sexual and reproductive health issues with their peers. A 20-year-old female vendor using artificial limbs to walk said:

‘I am a member of a WhatsApp group for disabled women. This is where we usually discuss various issues that affect us. In my group there are some disabled women who are knowledgeable about sexual and reproductive health issues. I get most of the information on sexual and reproductive issues from my WhatsApp friends.’

Another social medium where women with disabilities get information on sexual and reproductive health is Facebook. This was also corroborated by a maid who is 26 years old female with a stammer:

‘Facebook has sites which one can like and follow. From these sites one can get general information on sexual and reproductive health. We also surf some information from Google and Wikipedia. The internet has helped us a lot though some information cannot be authenticated. We use our phones to connect to the internet. However, some of my friends cannot access the internet because they do not have phones that are compatible with internet.’

Friends are also a source of information on sexual and reproductive health for women with disabilities. Respondents highlighted that they ask their friends a lot about sexual and reproductive health. A 28-year-old female deaf participant said:

‘Our friends who appreciate our disabilities are always willing to educate us on a number of issues including sexual and reproductive health.’

Other social groupings such as church groups and social clubs are important sources of information on sexual and reproductive health. While women with disabilities belong to and benefit from inclusive social groupings, they expressed feelings of marginalisation in some of these groups.

A 39-year-old female airtime vendor participant who is calipered added:

‘I belong to a women’s group in our church in which we discuss a lot of things. I have realised, however, that when I am present members deliberately avoid discussing sexual and reproductive health issues.’

## Discussion

The findings of the research prove that women with disabilities face multiple challenges in their effort to reach sexual and reproductive health services. As noted by various authors, this has become a global challenge (Mprah 2013). Negative attitudes still prevail the world over. Access to information is vital in making decisions, especially in the area that concerns one’s health. Women with disabilities, especially the deaf community, lack access to information. Evidence from this study demonstrates that women with disabilities have little access to formal information. Their right to information is restricted. Mprah (2013) in his studies on the deaf women in Ghana found out that the chief barrier to accessing sexual and reproductive health was the restricted access to information. There are many barriers faced by women with disabilities in trying to get access to sexual and reproductive health information and services. Deaf women also pointed out that they do not have access to informative information. This was also supported by women who are visually impaired who highlighted the unavailability of Braille information on sexual and reproductive health.

Policy inclusion is another good alternative of guaranteeing access to everyone. When enacting policies, government and development partners should thrive to include issues that affect people with disabilities. In a study done by Ahumuza et al. (2014), it was found that in Uganda, issues that affect women with disabilities with regard to getting access to sexual and reproductive health are well articulated in the policy. This had allowed health service providers to conform to the national policy. The Zimbabwean policy on sexual and reproductive health does not capture issues that affect women with disabilities. The UNCRPD (2007) recognised the challenges faced by women with disabilities and called for governments to include people with disabilities when enacting sexual and reproductive health.

Physical challenges continue to pose a challenge to women with disabilities when visiting health centres. The UNCRPD (2007) had called on the government to build buildings with ramps and elevators so that women with disabilities could gain easy entrance. In Zimbabwe, women with physical challenges highlighted physical barriers and clean toilets as a hindrance when visiting health facilities. These physical hindrances are also compounded by the attitudes of health workers at the health centres who are unable to understand the issues faced by people with disabilities. Evidence demonstrates a violation of privacy of women with disabilities. Health personnel do not understood sign language; however, a sizeable number of women who are deaf need access to primary health care. Awareness on issues that affect women with disabilities in accessing sexual and reproductive health care is also lacking.

## Recommendations

Women with disabilities are sexual beings. They need information and services for sexual and reproductive health for them to make informed choices and manage their sexuality and reproductive health needs. Government in partnership with other development agencies and the community should make efforts to improve the sexual and reproductive rights of women with disabilities. Based on the findings of this study, we make the following recommendations:

- The government, in partnership with other stakeholders, should address challenges faced by women with disabilities when accessing sexual and reproductive health services. Non-government, private hospitals and profit-making organisations should join hands with government in funding health requirements for women with disabilities. As enshrined in the national constitution, the government and other development partners should work hard to guarantee access to sexual and reproductive health for women with disabilities.
- Infrastructure must be improved to suit the needs of people with disabilities, particularly women with disabilities. Ramps and disabled-friendly toilets must be constructed at all public places, especially hospitals and clinics. When constructing new clinics and hospitals, it should be recommended that these new places be disabled-friendly.
- Health training institutions and other public institutions should teach sign language to their staff to be able to interact with the deaf. A mechanism needs to be put in place to introduce sign language.
- Consultation fees need to be lowered to allow women with disabilities to reach health facilities.
- More information platforms for women with disabilities should be established. The government should fund programmes that particularly target deaf women so that they can also get information about their health.
- Organisations of women with disabilities must be capacitated to offer information on sexual and reproductive health.
- Political participation of women with disabilities must be encouraged. Thus, political parties should be encouraged to embrace women with disabilities within their political structures. Once they are politically active, they can better voice the issues that affect them.
